# Breedbase: a digital ecosystem for modern plant breeding

**DOI:** 10.1093/g3journal/jkac078

**Published:** 2022-04-06

**Authors:** Nicolas Morales, Alex C Ogbonna, Bryan J Ellerbrock, Guillaume J Bauchet, Titima Tantikanjana, Isaak Y Tecle, Adrian F Powell, David Lyon, Naama Menda, Christiano C Simoes, Surya Saha, Prashant Hosmani, Mirella Flores, Naftali Panitz, Ryan S Preble, Afolabi Agbona, Ismail Rabbi, Peter Kulakow, Prasad Peteti, Robert Kawuki, Williams Esuma, Micheal Kanaabi, Doreen M Chelangat, Ezenwanyi Uba, Adeyemi Olojede, Joseph Onyeka, Trushar Shah, Margaret Karanja, Chiedozie Egesi, Hale Tufan, Agre Paterne, Asrat Asfaw, Jean-Luc Jannink, Marnin Wolfe, Clay L Birkett, David J Waring, Jenna M Hershberger, Michael A Gore, Kelly R Robbins, Trevor Rife, Chaney Courtney, Jesse Poland, Elizabeth Arnaud, Marie-Angélique Laporte, Heneriko Kulembeka, Kasele Salum, Emmanuel Mrema, Allan Brown, Stanley Bayo, Brigitte Uwimana, Violet Akech, Craig Yencho, Bert de Boeck, Hugo Campos, Rony Swennen, Jeremy D Edwards, Lukas A Mueller

**Affiliations:** Boyce Thompson Institute, Ithaca, NY 14853, USA; Cornell University, Ithaca, NY 14853, USA; Boyce Thompson Institute, Ithaca, NY 14853, USA; Cornell University, Ithaca, NY 14853, USA; Boyce Thompson Institute, Ithaca, NY 14853, USA; Boyce Thompson Institute, Ithaca, NY 14853, USA; Boyce Thompson Institute, Ithaca, NY 14853, USA; Boyce Thompson Institute, Ithaca, NY 14853, USA; Boyce Thompson Institute, Ithaca, NY 14853, USA; Boyce Thompson Institute, Ithaca, NY 14853, USA; Boyce Thompson Institute, Ithaca, NY 14853, USA; Boyce Thompson Institute, Ithaca, NY 14853, USA; Boyce Thompson Institute, Ithaca, NY 14853, USA; Boyce Thompson Institute, Ithaca, NY 14853, USA; Boyce Thompson Institute, Ithaca, NY 14853, USA; Boyce Thompson Institute, Ithaca, NY 14853, USA; Boyce Thompson Institute, Ithaca, NY 14853, USA; IITA Ibadan, 200001 Ibadan, Nigeria; IITA Ibadan, 200001 Ibadan, Nigeria; IITA Ibadan, 200001 Ibadan, Nigeria; IITA Ibadan, 200001 Ibadan, Nigeria; NaCCRI, Namulonge, Uganda; NaCCRI, Namulonge, Uganda; NaCCRI, Namulonge, Uganda; NaCCRI, Namulonge, Uganda; National Root Crops Research Institute (NRCRI), 463109 Umudike, Nigeria; National Root Crops Research Institute (NRCRI), 463109 Umudike, Nigeria; National Root Crops Research Institute (NRCRI), 463109 Umudike, Nigeria; IITA Nairobi, 30709-00100 Nairobi, Kenya; IITA Nairobi, 30709-00100 Nairobi, Kenya; Boyce Thompson Institute, Ithaca, NY 14853, USA; IITA Ibadan, 200001 Ibadan, Nigeria; National Root Crops Research Institute (NRCRI), 463109 Umudike, Nigeria; Cornell University, Ithaca, NY 14853, USA; IITA Ibadan, 200001 Ibadan, Nigeria; IITA Abuja, 901101 Abuja, Nigeria; Cornell University, Ithaca, NY 14853, USA; USDA-ARS, Ithaca, NY 14853, USA; Cornell University, Ithaca, NY 14853, USA; Cornell University, Ithaca, NY 14853, USA; USDA-ARS, Ithaca, NY 14853, USA; Cornell University, Ithaca, NY 14853, USA; USDA-ARS, Ithaca, NY 14853, USA; Cornell University, Ithaca, NY 14853, USA; Cornell University, Ithaca, NY 14853, USA; Cornell University, Ithaca, NY 14853, USA; Kansas State University, Manhattan, KS 66506, USA; Kansas State University, Manhattan, KS 66506, USA; Kansas State University, Manhattan, KS 66506, USA; Bioversity-CIAT Alliance, 34397 Montpellier, France; Bioversity-CIAT Alliance, 34397 Montpellier, France; TARI, 33518 Ukiriguru, Tanzania; TARI, 33518 Ukiriguru, Tanzania; TARI, 33518 Ukiriguru, Tanzania; IITA Ibadan, 200001 Ibadan, Nigeria; IITA Ibadan, 200001 Ibadan, Nigeria; IITA Ibadan, 200001 Ibadan, Nigeria; IITA Ibadan, 200001 Ibadan, Nigeria; North Carolina State University (NCSU), Raleigh, NC 27695, USA; CIP, 15000 Lima, Peru; CIP, 15000 Lima, Peru; KU Leuven, 3000 Leuven, Belgium; USDA-ARS, Stuttgart, AR 72160, USA; Boyce Thompson Institute, Ithaca, NY 14853, USA

**Keywords:** database, breeding, phenotyping, genotyping, predictive breeding, genomic selection, genome-based breeding, digital ecosystem, digital agriculture, web-based software, open source breeding software

## Abstract

Modern breeding methods integrate next-generation sequencing and phenomics to identify plants with the best characteristics and greatest genetic merit for use as parents in subsequent breeding cycles to ultimately create improved cultivars able to sustain high adoption rates by farmers. This data-driven approach hinges on strong foundations in data management, quality control, and analytics. Of crucial importance is a central database able to (1) track breeding materials, (2) store experimental evaluations, (3) record phenotypic measurements using consistent ontologies, (4) store genotypic information, and (5) implement algorithms for analysis, prediction, and selection decisions. Because of the complexity of the breeding process, breeding databases also tend to be complex, difficult, and expensive to implement and maintain. Here, we present a breeding database system, Breedbase (https://breedbase.org/, last accessed 4/18/2022). Originally initiated as Cassavabase (https://cassavabase.org/, last accessed 4/18/2022) with the NextGen Cassava project (https://www.nextgencassava.org/, last accessed 4/18/2022), and later developed into a crop-agnostic system, it is presently used by dozens of different crops and projects. The system is web based and is available as open source software. It is available on GitHub (https://github.com/solgenomics/, last accessed 4/18/2022) and packaged in a Docker image for deployment (https://hub.docker.com/u/breedbase, last accessed 4/18/2022). The Breedbase system enables breeding programs to better manage and leverage their data for decision making within a fully integrated digital ecosystem.

## Introduction 

Modern plant breeding is a data-intensive process requiring multiple diverse datasets to be integrated and assessed in decision making. In classical plant breeding, promising individuals are intentionally interbred to generate a diverse population of progeny, from which individuals with the best phenotypic characteristics are selected to be used as elite parents in subsequent breeding cycles or released as improved cultivars (Breseghello and Coelho 2013). Modern plant breeding extends classical breeding with the use of marker-assisted selection and genomic selections (GS) to augment phenotypic selection (Ribaut and Hoisington 1998). Furthermore, with the emergence of high-throughput phenotyping technologies as tools for breeding, the number of potential phenotypes to be tracked has vastly increased (White *et al.* 2012; Andrade-Sanchez *et al.* 2013).

The development of inexpensive genotyping technologies allow even small breeding programs to acquire high-density genotyping data for a large portion of their germplasm. The availability of this genomic data has enabled more efficient approaches to evaluate important and complex traits in the breeding process (VanRaden 2008). One such approach is GS, which combines genomic and phenomic data to develop a predictive model that can be used to estimate genotypic or breeding values (Meuwissen and Goddard 2001). Since genotyping is both less expensive and faster than phenotypic selection, GS can result in significant acceleration of the breeding cycle with concomitant faster increases in gain. A challenge for genome-based breeding methods is the establishment of an adequate data management infrastructure to integrate the complex datasets spanning the breeding process (Volk *et al.* 2021). This represents a severe constraint to mainstreaming predictive breeding to small breeding programs, particularly in developing countries.

To address these data management challenges, we initiated a system called Cassavabase (https://cassavabase.org/, last accessed 4/18/2022) for the NextGen Cassava project building on a genomics codebase developed for many years for the Solanaceae called SGN (https://solgenomics.net/, last accessed 4/18/2022) (Mueller, Solow, *et al.* 2005; Menda *et al.* 2008; Bombarely *et al.* 2011; Fernandez-Pozo, Menda, *et al.* 2015). With an initial focus on tomato and sequencing its genome (Mueller, Tanksley, *et al.* 2005; Tomato Genome Consortium 2012), SGN already contained a comprehensive genomics database with a strong phenotype management component (Menda *et al.* 2008), a number of genomics-centric tools (Mueller *et al.* 2008; Tecle *et al.* 2010; Fernandez-Pozo, Rosli, *et al.* 2015), and a rudimentary version of a genotyping storage backend (Fernandez-Pozo, Menda, *et al.* 2015). Cassavabase is an open-source, web-based breeding data management and analysis system built with the ability to manage the GS process (Tecle *et al.* 2014). As more instances of the software were deployed for other crops, the system expanded to better meet each project’s needs by adding further breeding-related tools, such as image-based or near-infrared spectroscopy (NIRS)-based phenotyping tools (Hershberger *et al*. 2021). To reflect that the underlying software and database are amenable to any crop and to promote adoption by new communities, we named the system “Breedbase” (https://breedbase.org/, last accessed 4/18/2022). Major clonal crops using Breedbase currently are cassava (https://cassavabase.org/, last accessed 4/18/2022), yam (https://yambase.org/, last accessed 4/18/2022), banana (https://musabase.org/, last accessed 4/18/2022), and sweetpotato (https://sweetpotatobase.org/, last accessed 4/18/2022), collectively known as the RTBbases (https://rtbbase.org/, last accessed 4/18/2022); however, major nonclonal crops using Breedbase include wheat (https://wheat.triticeaetoolbox.org/, last accessed 4/18/2022) and rice (https://ricebase.org/, last accessed 4/18/2022). Breeding and research groups have adopted the system as well, such as the Gore Lab at Cornell University (https://gorelabbase.sgn.cornell.edu/, last accessed 4/18/2022).

The purpose of Breedbase is to enable a *digital ecosystem* that contains an integrated breeding workflow. Processes and data comprising germplasm banks, parental selection, crossing design, experimental design, data collection, analyses, and decision-making tools are aggregated into a single system. This improves efficiency and reduces data errors that can happen when using disjointed informatics tools, for instance when transferring and restructuring data for analyses (Cobb *et al.* 2019). When data are loaded into a database, many checks can be performed to make sure the data are consistent and in line with specified quality control criteria.

Many breeders, especially in smaller programs that cannot allocate resources to data management tools, maintain their data in spreadsheets. While spreadsheets provide a straightforward way to manage data and analyses, they suffer from a number of drawbacks, even with relatively small volumes of data. For example, it is difficult to precisely merge data across different spreadsheets, often resulting in errors and data quality issues, or to visualize or analyze data across spreadsheets. Data in spreadsheets are typically not normalized, resulting in typographical issues, inconsistent identifiers, liberal use of synonyms, and similar issues that make the data hard to aggregate. Nevertheless, the largest problem with spreadsheets is that their storage is not centralized; in fact, they are often stored on personal computers and laptops, often in multiple inconsistent versions, with potentially limited backup strategies and little recourse if accidental data loss occurs or if a person leaves the breeding program, taking all the breeding data with them. Breeding programs can be very large, encompassing many locations with many collaborators; as such, spreadsheets hinder collaboration because data cannot be accessed in a consistent state by many people at once. Furthermore, with genome-based breeding, spreadsheets become unworkable, as it is difficult to maintain and analyze potentially very large genotypic datasets in spreadsheets in any useful way. It is important to note that using a database is not sufficient for managing a modern breeding cycle—the entire breeding process needs to be integrated around the database to create an efficient digital ecosystem.

Breedbase implements a robust system of breeding workflows, data management procedures, and analysis tools to address breeder informatics problems. Here we present the rationale, design, implementation, and major use cases for Breedbase.

## Materials and methods

### Implementation

**Fig. 1.**
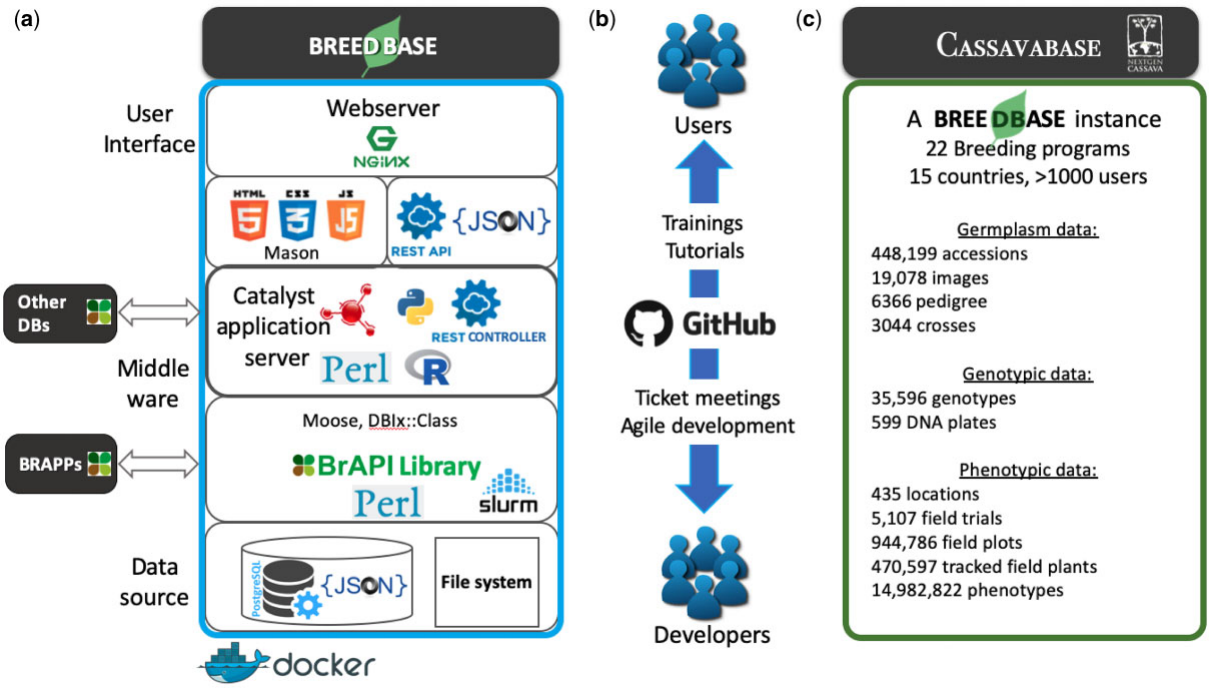
a) Breedbase platform architecture. User interface: To offer a dynamic, highly interactive user interface, several JavaScript libraries are implemented including D3, JQuery, and Bootstrap. RESTful APIs, including a full BrAPI 2.0 implementation, handle the communication between the front and back end, allowing fast calculations without reloading the website. HTML5 for interactive graphical display, allowing instant reorganization of visual elements. The Bootstrap framework is used for modern and dynamic page templating. Middleware layer: A Perl software stack including Mason components to connect to the user interface, a Catalyst a web application framework, Moose an object oriented perl library and DBIX::Class an object-relational mapper to connect to SQL code. In addition, BrAPI libraries are used. Finally a job cluster scheduler, Slurm is implemented to allocate server resources and ensure scalability. Data source layer: Breedbase operates on a relational database using Postgres. Postgres 12.0 offers “Big data” solutions including parallel query execution and optimized binary JSON data type handling. Binary JSON (JSONB) is a simple data structure designed to be storage space and scan-speed efficient. In Breedbase, JSONB is used in various data types including genotypic (marker) information. In addition to the relational database a standard file system space is available for flat files. Finally, other databases can communicate to a Breedbase instance to provide additional back-end for marker data [i.e. Genomic Open Source Informatic Initiative (GOBii)] or to exchange germplasm information for example. b) Breedbase codevelopment process. User–developers interactions are promoted using various media. Users have online access to documentation (https://solgenomics.github.io/sgn/, last accessed 4/18/2022), video tutorials, or through onsite training. Software development goals are extensively discussed between developers, data managers, breeders, and other appropriate stakeholders. Agile development allows short-term product release. Suggested improvements, issues, and bugs discovered in Breedbase are submitted and tracked on the public GitHub issue tracking software (https://github.com/, last accessed 4/18/2022). Software development progress is tracked using a version control system and Docker releases. c) Cassavabase, a breedbase instance: data content overview. Cassavabase involves national and international breeding programs (22) from various African and South American countries (15) and currently has 1,131 registered users. Cassavabase hosts various data types including high-density and low-density genotyping assays (35,000), plot-based phenotypic data points (near 15 million), images from plants and plots from trials (5107) and locations (435).

The Breedbase data architecture is built around a Postgres (https://postgresql.org/, last accessed 4/18/2022) relational database with a schema that is mainly derived from Chado (Jung *et al.* 2011), with some historic, pre-Chado tables from SGN, as well as minor customizations (Fernandez-Pozo, Menda, *et al.* 2015) (Fig. 1a). In relational databases, information is systematically structured into concepts represented as tables (“normalization”), a format that facilitates many aspects of data management. The information in the different tables can be joined based on primary and foreign keys, which are usually numeric values assigned to every row in a table. For some data types, such as genotypic data, Breedbase uses non-SQL extensions built into Postgres, such as JSONb-based data structures (Morales, Bauchet *et al.* 2020). The application layer is implemented in Perl, using the Moose object system, based on the Model-View-Controller Catalyst web framework (https://metacpan.org/pod/Catalyst::Manual, last accessed 4/18/2022), with Mason as the templating toolkit (https://metacpan.org/pod/Mason). The system uses an object-relational layer based on DBIx::Class, with the main Chado classes organized in the Bio::Chado::Schema namespace. For statistical analyses and some of the data visualizations, the R language and add-on R packages (https://r-project.org/, last accessed 4/18/2022) are used. Image analyses and machine learning models are implemented in Python TensorFlow (https://www.tensorflow.org/, last accessed 4/18/2022 ) and OpenCV (https://opencv.org/, last accessed 4/18/2022) (Morales, Kaczmar, *et al.* 2020). The frontend graphical user interface (GUI) development has recently transitioned away from Mason components to JavaScript, with a heavy reliance on asynchronous JavaScript requests. Almost all functionalities are implemented as RESTful services, allowing for a more interactive user experience and reusable codebase. JavaScript frameworks used for the GUI include JQuery (https://openjsf.org/, last accessed 4/18/2022), D3.js (https://d3js.org/, last accessed 4/18/2022), Bootstrap (https://getbootstrap.com/, last accessed 4/18/2022), and Brapi.js (https://brapi.org/, last accessed 4/18/2022). The entire Breedbase system is built on open source software and is packaged in a Docker image for deployment (https://docker.com/, last accessed 4/18/2022). For interoperability with other breeding database and tools, Breedbase implements the BrAPI 2.0 specification (Selby *et al.* 2019).

In terms of user interface, the goal of Breedbase is to provide a standard, modern web interface for all breeding tools. Breedbase is essentially a cloud-based app, obviating the need for the user to install any software. For anyone with web-browsing experience, the interface should be intuitive and straightforward, and it is continuously improved based on user-driven feedback. In Breedbase, processes are presented in an interactive workflow system, providing step-by-step guidance to breeders and users in accomplishing specific tasks. A few of the widely used interfaces include the Wizard, Lists, and Datasets tools, which will be described in more detail later.

### Use cases

The initial development of BreedBase focused on addressing the data collection and management stages necessary to facilitate GS within a breeding program, including:

- Manage accessions and pedigrees in the database, with ontology-based descriptions and support for rich metadata including images
- Design field layouts and track all field metadata
- Load historical data from breeding programs
- Collect phenotypic data on tablets in the field and upload the subsequent phenotypes
- Manage genotypic data associated with the accessions
- Enable genome-based predictive breeding by calculating correlations between phenotypes and genotypes, and predict phenotypes from genotypes [the solGS tool (Tecle *et al.* 2014), https://cassavabase.org/solgs/search, last accessed 4/18/2022]
- Support controlled crossing using customized tracking tools

More recently, a number of other use cases were pursued:

- Advanced statistical analyses including principal component analysis (PCA), stability analysis (AMMI) (Duarte and Pinto 2002) heritability calculations (Holland *et al.* 2010), mixed model analysis, and genome-wide association studies (GWAS)
- Marker-assisted breeding
- Processing and analysis of unoccupied aerial vehicle image data
- Image analysis
- NIRS data storage and analysis

Plant breeding operations requiring decision support within a growing season include 3 broad activities: crossing, evaluations, and selections. These activities typically include setup of crossing and trial experiments (design, labeling), data and seed collection, genotyping, and subsequent statistical analysis. Breedbase offers support for each of these components through online tools. To streamline accessibility and usage for key routine activities, Breedbase has established workflow components. Each workflow offers the user a guided process for a targeted activity. For example, the trial creation workflow comprises trial creation, planting material and checklist creation, randomization and statistical design selection, field visualization, and storage. During this process, field trial experiment parameters (see Phenotyping Trials section) are input into Breedbase and the relevant experimental design is calculated using open source R libraries such as Agricolae (De Mendiburu *et al.* n.d.) or Digger (Coombes 2009). The experimental layout is calculated and displayed, and can be reviewed and potentially improved by rerunning randomization before the trial design is stored in Breedbase. Additional parameters such as field management factors (i.e. agronomic management or fertilizer application) can also be entered. Similar workflows exist for other activities, such as phenotyping and genotyping.

### Development process

The development process can be broadly described as agile (Beck and Andres 2004; James and Shane 2008), in which short-term goals are defined and implemented, and subsequently further refined based on new feedback from users; agile teams provide for short release cycles and continuous improvement to the software (Fig. 1b). Progress is tracked using a version control system with built-in issue tracking software (GitHub, https://github.com/). New features are discussed with breeders and other stakeholders. Issues and bugs discovered in Breedbase are tracked on the public GitHub issue tracker. A programmer is then assigned to a ticket, and will create an issue-specific topical git branch in the relevant code repositories, and implements the required changes in the branch, including tests and edits to the user documentation. When the implementation is ready for release, a pull request is generated on GitHub and a reviewer is assigned. In the review, the code is verified for errors, programming style, tests, and documentation. If the reviewer approves the pull request, the code is merged into the master branch. The test-driven software development approach is tightly integrated with our development process, consisting of unit and integration tests. A ticket meeting is held once a week and all open pull requests and important tickets are discussed. If all the pull requests were merged successfully, and no issues are discovered with tests or other checks, a new release tag is created, the new version is deployed in production, and a new Docker image is released. Since Breedbase is open source, programmers outside of the core development team are able to make contributions to the code base via the same process. The Breedbase project has had 40+ contributors addressing various issues and improvements (https://github.com/solgenomics/sgn/graphs/contributors).

### Ontologies

A key aspect of data integration is the necessity of standardization. Breedbase is based on the Chado database schema, which relies heavily on controlled vocabularies and ontologies to describe its data, and requires numerous ontologies for its internal functioning. In many ways, it can be described as an ontology-based database. For the breeding application, data standardization in the form of trait catalogs is especially important when several sites or breeding programs share data in the database. Without standardization, the data would not be comparable, limiting the utility of an integrated database. The creation and maintenance of trait ontologies is a considerable task. The Crop Ontology (CO) project was developed by CGIAR to define and maintain relevant breeding ontologies (Shrestha *et al.* 2012). All the RTBbases use the CO vocabularies and collaborate with CO and breeders to improve and expand these vocabularies (Arnaud *et al.* 2020). If no ontologies are available, they have to be created, which can be a lengthy and arduous task. The Protégé tool (https://protege.stanford.edu/) (Musen 2015) is commonly used by curators for editing ontologies before upload to CO and Breedbase. The Trait Dictionary Template along with the Guidelines (Pietragalla *et al.* 2020), available in the CO website, remain useful to collect the trait details from the research community and reach consensus. Each species is allocated a code by the CO coordination team to identify the ontology and crop repositories are created in the Planteome Github to secure the ontology version management. An online term submission form is accessible in Breedbase for users wishing to suggest missing traits or modifications to the CO (https://submit.rtbbase.org, last accessed 4/18/2022).

### Interoperability and BrAPPs

Databases must interoperate with a variety of tools to perform their functions in data acquisition, analysis, and data export. Recently, a standard called the Breeding Application Programming Interface (BrAPI; https://brapi.org/) was developed to exchange breeding data (Selby *et al.* 2019), which breeding databases can implement to provide a standard interoperability layer. Standardized application programming interfaces (APIs) allow Breedbase to integrate and interface with a broader set of BrAPI-enabled applications, or BrAPPs, that can be written across diverse programming languages including Android, R, and JavaScript. The BrAPI R package allows data retrieval from Breedbase for further statistical processing within the R environment. JavaScript-based BrAPPs provide dynamic visualization of plant breeding data, such as pedigrees exploration, experimental field maps, and data from multiple trials. BrAPPs can interact with data from any BrAPI compliant database, such as Breedbase or the Breeding Management System (Fig. 1a). Activities such as dynamic data filtering, trial comparison, box plotting, and a comparative genetic map viewer are also implemented with BrAPPs on Breedbase. Breedbase fully supports BrAPI version 2.0 and is committed to updating the system for future versions of this essential infrastructure.

#### Querying Breedbase

**Fig. 2.**
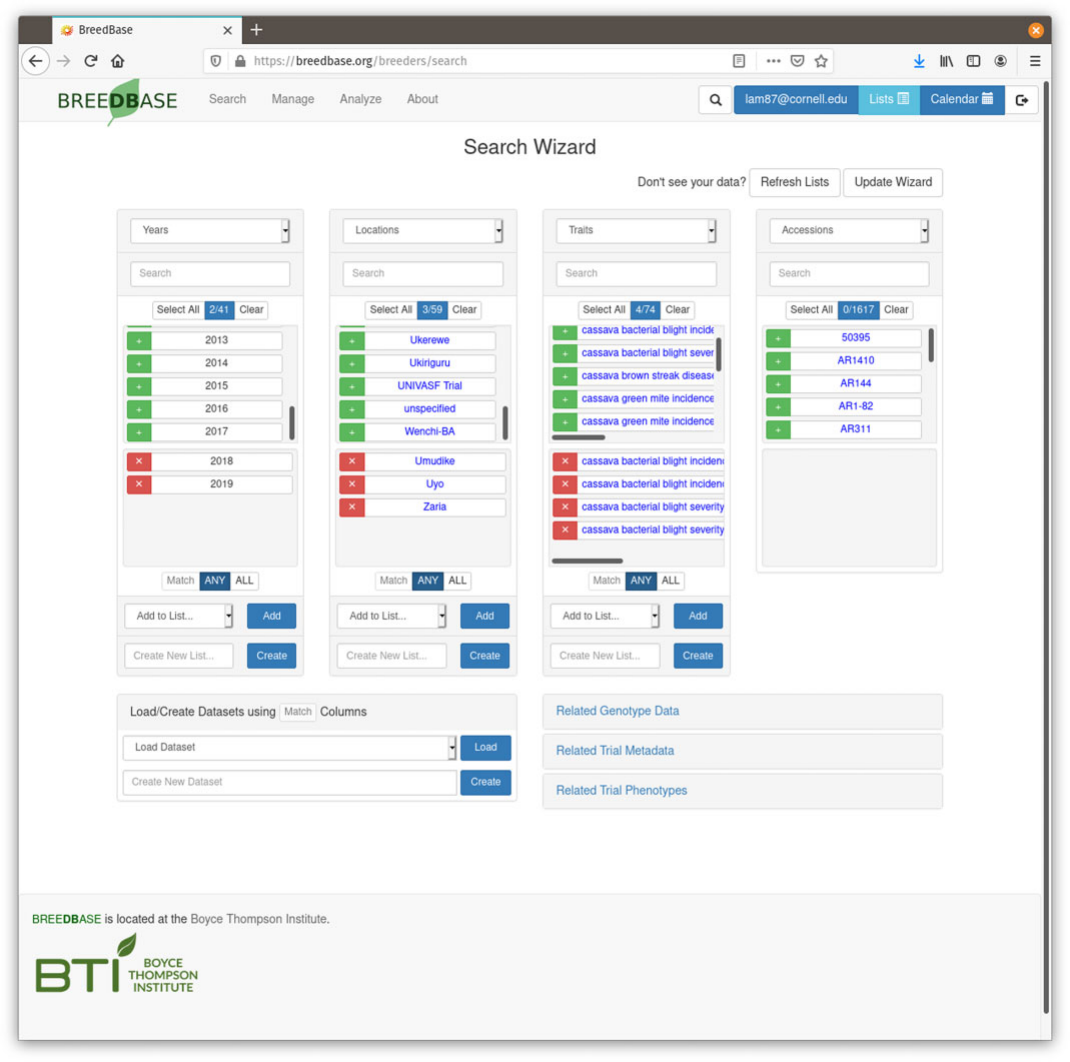
Screenshot of the “Search Wizard” interface, a central query function on Breedbase. With the Search Wizard, the data in the database can be intersected by dimensions, such as locations, years, breeding programs, and traits. For each dimension, a number of elements can be selected. The individual selected dimensions can be stored in lists, and the combined selections can be saved as a dataset. Both lists and datasets can be used to feed data into various tools on Breedbase.

Breedbase has a number of query options, which are grouped in the “Search” menu. The most important data types each have a search (“Accessions and plots,” “Trials,” “Organisms,” “Crosses,” etc.). A powerful combined search is available in the form of the Search Wizard (Fig. 2).

### The Search Wizard and datasets

The Search Wizard allows users to slice their data in different dimensions, such as breeding programs, locations, years, and so forth. The data in the database can be thought of as a multidimensional cube which is cut along different dimensions, providing an intersection that represents the data of interest. This approach is conceptually related to a query method called Online Analytical Processing (Celko 2006). The current Wizard presents 4 boxes, for 4 different dimensions, which can be selected using pull down menus (Fig. 2). For example, a user who is interested in the performance of cassava clones evaluated by IITA in 2017 and 2018 at the Mokwa station in Nigeria can use the wizard to find this information. Working from left to right, the user selects as the first dimension “Breeding Programs,” which displays all the breeding programs in the database in the first box. The user then selects “IITA” from the individual breeding programs listed in the box. When the user selects “Years” in the second box, all the years for which data for IITA exist are listed. In this example, the user selects 2017 and 2018. Finally, after selecting locations in the third box, the user specifies “Mokwa.” When trials or accessions are selected, phenotypic and genotypic data corresponding to the selection can be downloaded using buttons below the Wizard boxes. The Wizard also allows the combination of current selections to be stored in the database under a user-given name, representing an intersect of data of interest in the database. This stored selection is called a “dataset.” Datasets are used across Breedbase to efficiently reference a complex query with a simple, assigned name. Tools that support the dataset concept in Breedbase include solGS, GWAS, the heritability tool, the stability analysis, and the general mixed model tool.

### Quick search

A quick search is provided in the upper right corner of the menu bar that searches a keyword across all data types in the database, and is a fast way to retrieve named objects such as stocks and genes.

#### Special searches

Topic-specific searches are available from the Search menu, including a trial search, a trait search, searches for genotyping data (including genotyping protocols, projects, and plates), an image search that searches image descriptions and associated tags, and a user search that searches the users of the database. All these searches work in a straightforward and consistent way: a search form is filled in with search criteria, and the search is submitted to the database. A list with matched search results is displayed, from which links are provided to the corresponding detail pages.

### Analysis tools

Breedbase is more than a static collection of data, as it enables users to explore and analyze data in the database. Once data is uploaded to the database, users can view summary statistics, evaluate phenotypic variances, and identify observations with missing or outlier data. They can filter observations in a trial based on a range of trait or traits values. For an experiment phenotyped in multiple environments, they can evaluate trait performance across environments using pairwise comparison scatter plots and histograms.

Breedbase also has tools for ANOVA, correlation, PCA, data partitioning using K-means clustering, genomic prediction, GWAS, selection index calculation, genetic gain visualization, and linear mixed models. With the Search Wizard, as explained above, users can construct datasets that can be used as inputs to various tools. Most tools follow a similar blueprint in terms of user interface: (1) select the dataset of interest from a drop-down menu of all available datasets, (2) adjust parameters for the tool, (3) submit the calculation for analysis, and (4) display the results. For some tools that require heavy computation, an email can be optionally sent to the user with a link to the results. Query implementation is a relatively complex task in the programming of a tool, but the Wizard enables the modularization of algorithms into Breedbase with relatively little glue-code, facilitating tool coverage expansion. Results such as predictions from solGS and adjusted means from mixed models can be saved in the database as analysis results. These results can be used like primary data in downstream analyses such as the selection index tool to help identify favorable germplasm.

### Managing a breeding program using Breedbase: accessions, phenotyping, crossing, and genotyping data

#### General principles

Plant breeding involves the collection of a wide variety of data types at different time points and locations, and across different scenarios (e.g. field, laboratory, seed storage). To give users flexibility and mobility in data collection, smartphone-based applications are often required. Android applications, such as PhenoApps (http://phenoapps.org/, last accessed 4/18/2022), are developed with this perspective (Rife and Poland 2014). Breedbase has adopted the PhenoApps tool suite created by Kansas State University.

PhenoApps include applications for phenotyping (Field Book), cross management (Intercross), sample collection (Coordinate), and inventory management (Inventory). Breedbase has worked to build in native support for these applications and integrate them into best practices workflows. Since internet access is not available at all field sites, the functionality has been developed to allow configuration of these applications prior to field data collection. Field layouts, plant accessions, and traits to be measured can be loaded onto mobile devices through special interfaces in Breedbase. Following collection, data are imported back into Breedbase. Because all the trial information in the collection device was initially downloaded from Breedbase, required identifiers can easily be matched with the existing data in the database. This process is called “round-tripping,” and is a crucially important concept for high quality data management.

#### List management

Breeding activities often require the maintenance of lists of various types—for example, a list of accessions to plant, traits to measure, or trials to evaluate—and, consistent with digital ecosystem principles, these lists should be managed entirely through the database. Accordingly, Breedbase implements comprehensive list management functions. By default, lists are associated with the user that creates the list. The main list interface can be reached by clicking on the Lists link on the top right of the toolbar, which appears when logged in. A dialog appears that allows users to view, create, and edit new lists. Each list has a data type from an internal ontology called “list_type,” which includes terms for “accessions,” “trials,” “traits,” “years,” etc. Lists are collections of text elements that correspond to names of database objects. Lists can be validated against names that are already present in the database. A validated list can then be used to submit data to various tools, including the Wizard, right on the website. Sometimes, it can be useful to share a list with other users, and this can be achieved by making a list public by clicking the appropriate checkbox in the list detail view. Public lists are shown in a separate section, and become visible to all users. They can be “unshared” if needed.

#### Germplasm management

Germplasm is the foundation of a breeding program and plays a similarly important role in a database such as Breedbase. In plant breeding programs, tracking and characterization of germplasm is a major challenge. Germplasm in this context includes accessions, stocks, varieties, or, in clonal crops, clones. Breedbase commonly uses the term “accession.” Breedbase is prepopulated with the complete plant section of the NCBI taxonomy database, defining all known species with their associated genus, abbreviation, common name, and GenBank taxon identifier. Researchers using Breedbase can usually find their crops of interest within the 100,000+ organisms available. Accessions are always created in association with one of these organisms.

Some instances of Breedbase, such as Cassavabase and Sweetpotatobase, are designed to only contain germplasm of their respective species; however, it is possible for a single instance of Breedbase to be used for a variety of crop species. Combining many crop species into a single instance can complicate the search interfaces and lead to bloated databases; however, aggregating all data allows for more consistent and queryable data. Alternatively, separating instances can lead to potentially duplicated and inconsistent data, but can be beneficial for fostering communities.

In Breedbase, there are 2 distinct concepts that describe accessions: (1) an accession that can be ordered from a seed bank, which may have been selfed and could be genetically quite pure, or landraces. These are “long-term use” accessions (i.e. historical germplasm, parental inbred lines), which may be actively maintained and can be obtained easily; whereas (2), are “short-term use” accessions (i.e. intermediate generations) that are produced in a breeding program and may go through a few rounds of selection, but most of which will be discarded in the process. These accessions may also not be genetically pure, as they may result from crosses between relatively distant parents.

To create an accession in Breedbase, only a unique name and the organism species name are required. As with all objects stored in a relational database, Postgres will create a primary key identifier for each object, using a data structure called a sequence, which is used to link the accession to other objects in the database using a foreign key. This means that even if the accession name is modified, it will still retain all the connections to other objects of the original entry. Germplasm can be further annotated with configurable properties from the Multi-Crop Passport Descriptors standards (Food and Agriculture Organization of the United Nations 2018) and BrAPI standards (Selby *et al.* 2019); these properties include “variety,” “donor,” “donor institute,” “donor PUI,” “country of origin,” “institute code,” “institute name,” “notes,” “accession number,” and “PUI.” Germplasm can be added to the database using the interactive list tool (see previous section) or an Excel file upload; the Excel file upload also allows for storing and updating of all attributes listed above. The first step in the initiation of a breeding program is to load relevant accessions into the database. This is critical, as the naming of accessions is often not uniform between breeding programs and the community at large. In some cases, a single name can refer to several different accessions or a single accession may have many different names or synonyms, often the result of historical transcription error or case inconsistency. Before the first upload, it is therefore essential to define a standard unique name and set of possible synonyms for each accession. Though Breedbase allows for synonyms of accession names, they should also be unique. It is best practice to use synonyms only to find accessions and not when performing routine tasks with the database during the breeding process. Whenever new accession names are encountered, Breedbase provides a workflow to compare new names to all existing accessions in the database. In this workflow, a user can consolidate synonyms, for instance to add “Tx 303” as a synonym of “TX303.”

After initial accession upload, it is often necessary to add more accessions, increasing the chance of generating duplicated accessions in the database, or other upload issues. As is the case with synonyms, many of these problems result from poorly defined accession identifiers with capitalization inconsistencies and special characters such as slashes, dots, dashes, underlines, and spaces. Although we recommend avoiding such special characters, especially in primary identifiers, it is not always feasible, notably with legacy data. To ease upload and tracking of such cases, Breedbase has a fuzzy search (also called approximate string matching search) component, enabling an accurate quality control of existing similar germplasm names in the database.

#### Phenotyping trials

Phenotyping trials are a core activity of plant breeding programs, and must be carefully designed. Trial designs can either be generated directly in Breedbase using the integrated, comprehensive trial design tool or uploaded using Excel files formatted with a Breedbase-provided template. Trial metadata fields include breeding program, location, name, trial type, year, plot dimensions, field size, and trial design type. Supported statistical trial design types currently include alpha lattice, lattice, augmented, split plot, partially replicated, and Wescott designs. Designs should also include the ordinal row and column positions of each plot as it is planted in the field, so Breedbase allows this information to be added either during or after design storage. Once a trial design is finalized, it is stored in the Breedbase schema. Within Breedbase, a field trial links phenotypic observations to the experimental layout under a specific statistical design.

Row crops usually use the concept of plot as the minimal entity for data collection, but many specialty crops (i.e. vegetables) require data collection on a per plant or per tissue basis. Breedbase allows plant- and tissue-level entry creation for each plot in a trial, resulting in database entries and identifiers at each level, which can also be encoded in barcode labels for data collection.

#### Crossing

To collect data from crosses, Breedbase requires the creation of a top-level crossing experiment; the crossing experiment is defined with a unique name, a breeding program, a location, a year, and a description. The individual crosses performed are then stored under the crossing experiment and defined by a cross unique id, parents, and a cross type. The cross type can be one of the following: biparental, self, sib, open pollinated, bulk, bulk selfed, bulk and open-pollinated, doubled haploid, polycross, reciprocal, or multicross. Depending on the type of cross performed, different metadata must be provided; for example, in a biparental cross, information from both the male and female parent is required, whereas in an open-pollinated cross, information on only the female is required. In the case of an open-pollinated cross, a population name representing a group of male germplasm can be given as the male parent. In addition to cross unique id, which captures specific details of each cross, users have the option to group crosses having the same parental genotypes via family name for downstream progeny analysis.

Breedbase tracks parental information from crosses in 2 ways: (1) through the accession names of the female and male parents, allowing for simple ancestry tracking of AxB pedigrees for the progeny from a cross. When a cross is created in Breedbase, the pedigree between progeny and parental germplasm is automatically created as well. This first form of parental tracking is applied in all cases when a cross is created in Breedbase. (2) Through the plot or plant names of the male and female parents. The plot or plant names of the parents are related to the field trial in which they are planted, as is described in the above field trial section. This approach allows detailed tracking of female and male parents used in crossing, but is optional in Breedbase because of the difficulty in recording this information in many cases.

Recording information on parental plots is facilitated by mobile data collection platforms. Of note are customized Open Data Kit (ODK) Android applications, such as BTract and the PhenoApps app Intercross. BTract assigns and prints a unique cross barcode label after scanning barcodes to track the precise male and female plots or plants involved in the pollination. Through ODK data synchronization, the cross information can be uploaded into Breedbase. Intercross can be used to scan parental barcodes and associate a unique cross id to the performed cross. The output from Intercross can also be uploaded directly into Breedbase.

In crossing experiments that include evaluation of crosses, Breedbase can store annotations regarding properties of the cross. Default properties include pollination date, tag number, number of flowers, number of bags, number of fruits, and number of seeds; however, these properties are set in the configuration file for the Breedbase instance, allowing researchers flexibility in defining these terms. Breedbase also supports tracking of tissue culture samples.

Crosses can be created individually using an interactive interface on Breedbase or can be uploaded in bulk using an Excel spreadsheet by providing cross unique ids, cross types, and parents involved. Once each cross unique id is saved in Breedbase, additional data can be added or uploaded using the cross unique id as an identifier. Progeny of the cross can be saved as new germplasm in the database, automatically creating pedigrees for the new germplasm.

#### Genotyping data

High-density genotyping data are a complex data type that have become an important resource in modern breeding programs due to the advent of low-cost next-generation sequencing and genotyping technologies (Thomson 2014). Breedbase offers simple laboratory information management functionalities from field tissue sampling to SNP data storage. Functions include tissue samples collection and tracking via plot barcodes and PCR plate formats (i.e. 96 or 384 wells), genotyping protocol definition, data storage, and subsequent analytics (Tecle *et al.* 2014; Morales, Bauchet, *et al.* 2020).

The primary means of organizing genotyping data between sequencing events is the “genotyping protocol” in Breedbase. A “genotyping protocol” consists of a specific set of genotypic markers and records all metadata about how the genotypes were produced, including the reference genome and specifics about, analytical platform and related variant calling software. The “genotyping protocols” can be grouped in Breedbase under a “genotyping project” which displays all relevant genotyping data and provides an overview, which is especially useful for very active genotyping programs.

Multiple genotyping technologies can be stored in Breedbase from low density genotyping (i.e. Kompetitive allele-specific PCR, KASP) to high density genotyping such as genotyping-by-sequencing or DArT-seq (Elshire *et al.* 2011; Kilian *et al.* 2012; Semagn *et al.* 2014). The preferred method for uploading high-density genotyping data to Breedbase is through variant call format (VCF) files. VCF provides for compact representation of genotypic scores for large numbers of samples and markers (Danecek *et al.* 2011). PostgreSQL nonrelational functionalities allow Breedbase to store high-density genotyping data in JavaScript object notation (JSON) structures within the larger relational database schema (ISO/IEC TR 19075-6:2017 2018). Breedbase particularly relies on the binary JSON (JSONb) data type for compressed data storage and faster retrieval (Morales, Bauchet, *et al.* 2020).

Genotyping data can be queried alongside relationally stored phenotypic and experimental information for analyses, including computation of a genomic relationship matrix for user specified germplasm and computation of a GWAS for user specified germplasm and phenotypic traits (VanRaden 2008). Queries spanning specific markers or marker sets and experimental information can be readily constructed. Genotyping data results can be downloaded as VCF files from the Search Wizard web interface. The genotyping data are also used in the Genomic Selection tool, solGS, to predict GEBVs of genotyped lines.

#### Authentication and authorization

During breeding processes, a potentially large number of people will need to access the database to download, upload, modify, or delete data. This requires a fine-tuned layer of authentication and authorization management in the database. Breedbase requires a user to login for most functionalities (authentication). Every user account is associated with “roles” that determine what the user will be allowed to do in the system (authorization). Currently, there are 3 major roles: user, submitter, and curator. The user role allows read-only access. With the submitter role, a user can upload data, and can modify or delete data that they themselves uploaded. The curator role allows a user to modify any type of data. In addition, every breeding program in the database has a corresponding role that controls authorization over specific breeding program activities, such as creating and uploading trial data.

#### Cassavabase, the flagship Breedbase database

Cassavabase (https://cassavabase.org/) is the breeding database for the NextGen Cassava project (https://nextgencassava.org/, last accessed 4/18/2022). The NextGen Cassava partners, IITA (Ibadan, Nigeria), NRCRI (Umudike, Nigeria), NaCRRI (Namulonge, Uganda), TARI (Ukiriguru, Tanzania), Embrapa (Cruz das Almas, Brazil), and CIAT (Cali, Colombia) use Cassavabase for their breeding programs, starting as early as 2014. To date, Cassavabase has accumulated an immense amount of cassava breeding data (Fig. 1c), consisting of information on more than 500,000 cassava accessions, characterized by over 19 million phenotypic measurements in over 4,000 trials, and nearly 35,000 genotyping experiments. This shows that the Breedbase system can scale to fairly large datasets and large, multi-institute and multinational programs.

#### Other instances of Breedbase

In addition to Cassavabase, Breedbase has been deployed for various crops, notably for other Roots, Tuber and Banana (RTB) crops (https://rtbbase.org/, last accessed 4/18/2022) in the CGIAR: banana, (https://musabase.org/, last accessed 4/18/2022), sweetpotato (https://sweetpotatobase.org/, last accessed 4/18/2022), and yam (https://yambase.org/, last accessed 4/18/2022). In addition, several dozen Breedbase instances are currently deployed for other crops, such as rice (https://ricebase.org/, last accessed 4/18/2022), wheat (https://wheat.triticeaetoolbox.org/, last accessed 4/18/2022), oat (https://oat.triticeaetoolbox.org/, last accessed 4/18/2022), kelp (https://sugarkelpbase.org/, last accessed 4/18/2022), potato, and maize. While the aforementioned projects use Breedbase for mainly breeding informatics purposes, other Breedbase instances focus on genomics. These include SGN (https://solgenomics.net/; Fernandez-Pozo, Menta, *et al.* 2015), which focuses on tomato and other *Solanaceae*, fern (https://fernabase.org/, last accessed 4/18/2022;Li *et al.* 2018), *Erysimum* (https://erysimum.org/, last accessed 4/18/2022; Züst *et al.* 2020), and milkweed (https://milkweedbase.org/, last accessed 4/18/2022). In addition, a Breedbase instance has been deployed to characterize a tritrophic vector-borne disease system, the citrus greening disease (https://citrusgreening.org/, last accessed 4/18/2022) (Saha *et al.* 2017). An instance named ImageBreed has been deployed for high-throughput imaging of maize and alfalfa field experiments (https://imagebreed.org/, last accessed 4/18/2022; Morales, Kaczmar, *et al.* 2020). A number of academic labs and breeding companies also use Breedbase for data management within their programs. The Breeding Insight project (https://breedinginsight.org/, last accessed 4/18/2022), which creates breeding databases for USDA breeding programs, has also adopted the Breedbase system as a foundation for their breeding solutions.

**Box 1. Providing data management tools for small grains breeders: the Triticeae Toolbox adaptation of Breedbase**

As documented in this article, Breedbase provides many features for working breeding programs. The mission of The Triticeae Toolbox (T3) is to provide these features to a diverse audience of small grains breeding programs, by mandate in the United States, and by extension globally.

The development of T3 is motivated by the belief that larger datasets provide greater power to identify genetic effects that are relevant to all breeders. Across wheat, oat, and barley, T3 stores 5,600 trials, comprising over 1,800,000 phenotypic data points on over 30,000 lines with genotype data. From there, T3 seeks to provide breeders with results from analyses that tap into these data, in the hope that this will help breeders gain insights from their own data. The primary example we have in this area is a function to show marker trait associations identified among all trials submitted to T3 with adequate marker density, and meta-analyzed to determine robust associations across trials. The next milestone on the roadmap of this function is to develop marker imputation functionality on T3 that will present genotype trials with uniform high-density marker scores, enabling meta-analysis over more trials. Indeed, marker data are a critical rationale for T3’s mission: the database contains data on many lines that now are connected to current populations primarily through the marker alleles segregating.

An important advantage of a web-based data management platform is that it links the data to the world of knowledge available on the web. T3 provides that connectivity by providing links to external information on markers, traits, and germplasm. Our primary partners in that regard are GrainGenes, Wheat Expression Browser, and the Wheat KnetMiner (Hassani-Pak *et al.* 2021). For example, a marker trait association close to a gene can be used to connect that trait to JBrowse (https://jbrowse.org/, last accessed 4/18/2022), to gene expression data (expVIP and EMBL-EBI) or to a knowledge network, KnetMiner. Traits in Breedbase are defined using collaborative ontologies crucial to forging these links: the ontologies represent agreements on naming traits and gene functions that enable meaningful bridges across knowledge platforms.

The diversity of T3 users means that they will not operate together as an integrated breeding organization. Rather each breeding program submitting data to T3 will want data privacy and ease in determining what data becomes incorporated into the public production database. Currently, all data on the production database is available to anyone. We plan on implementing privacy settings specifying data visibility as public or restricted. Absent this feature, we now work with a few users by providing them with separate instances of T3 that are not publicly visible but can easily transmit datasets to the T3 production database when ready.

The wide range of T3 users also means that we expect them to have varying degrees of familiarity with the Breedbase platform. To allow users to test the addition and modification of datasets without modifying curated data by mistake, T3 has created sandbox instances for each crop. Users can freely upload data to the sandbox, ensure that the uploaded data added to the database is correct, and then easily publish the data to the production instance. A data curator checks the submitted data before adding it to the production database. The Breedbase system was crucial in establishing these features and reduced duplication of effort.

**Box 2. Usage example**

Recently, Ogbonna *et al.* (2021) leveraged legacy breeding data to investigate the genetic architecture of cyanide content in cassava, a key trait in food safety. Authors performed a retrospective analysis, mining historical cyanide data from the African IITA breeding program (18 locations, 23 years, and 393 trials) and Colombian CIAT (41 locations, 11 years, and 155 trials) program from the Breedbase instance cassavabase.org. Recycling open source, standardized, breeding data in conjunction with novel genotypic data provided a high statistical power and allowed the detection of key loci controlling cassava root cyanide content using GWAS. Such loci would otherwise have gone undetected, and was identified only because of the availability of the Breedbase digital ecosystem.

## Discussion

Breeding is a complex process involving many different types of data, especially considering genome-based breeding methods at the current state of the art. Creating and maintaining breeding databases is therefore generally considered to be time-consuming and expensive. Many large breeding companies maintain their own databases and software for managing breeding processes and selection, but this is not an option for smaller programs. The lack of bespoke databases is especially true in resource poor areas of the world, where the need for plant improvement is often the greatest. A free, user-driven, and open source platform such as Breedbase that integrates a complete digital ecosystem for breeding will help close the gap for these programs as well as many smaller to mid-sized organizations. Still, Breedbase databases can scale significantly to large breeding programs with hundreds of thousands of accessions and millions of phenotypic scores.

### Integration in breeding programs

Even the best breeding data management tools will fail to deliver if breeding programs do not use them or use them incorrectly. A significant effort is required to integrate a breeding database into the workflow of a breeding organization, as data management is central to the work of modern breeding programs but remains a shortcoming. Breeding activities need to be closely tracked; to ensure complete integration, all materials, operations, and operators need to be systematically recorded and reviewed throughout the process. This is important to enable analyses, improve data quality, and to identify sources of errors in real time and post hoc.

It is important for breeding programs to work closely with groups that have significant experience in data management, which can also help the breeding programs to understand their needs, and to train staff better in the use of the database. In the RTB breeding programs, we found it to be helpful to designate specific staff as Data Managers, who receive extensive database training. Data Managers have spent time at the BTI to learn more about the database developments, and can provide additional training and help on the ground in the breeding programs. They also provide timely feedback on the tools and features based on their first hand experiences, which is vital for the improvement of the database. We have put a significant effort in user training through in-person workshops, reciprocal visits, and training materials, such as a complete on-line manual, slideshows, and most recently a YouTube channel with recorded workshops.

In our experience, one of the bottlenecks in implementing a breeding database is the availability of standardized trait ontologies for the crop in question. Especially in larger projects, it can be difficult for all breeders to agree on a common ontology, including common sample preparation and measurement protocols, as well as measurement units. Without this standardization, a database loses much of its appeal as it becomes impossible to aggregate and reconcile disparate data. This challenge cannot be understated as it is a major obstacle especially when phenotypic data is collected across different locations for a variety of crops and has to be stored in a single integrated system. We have focused on developing ontologies and common vocabularies to address this issue but it can be harder than expected, as there are often diverging and strong opinions on these matters. In addition, breeding programs introduce new traits to be measured, for example, quality traits, and there needs to be a process to integrate such new terms into the ontology. Fortunately, the CO project (Shrestha *et al.* 2012) has created trait ontologies for a wide range of crops, which we contribute to and many Breedbase instances rely on. CO has also defined processes for updating and developing the ontologies, which allows new traits and methods to be introduced to breeding programs with relative ease.

### Future developments

Progress in the last few years in digital agriculture has been enormous and will continue to be so in the foreseeable future. New genotyping and phenotyping technologies, such as NIRS, are constantly being developed or improved. Breeding databases must coevolve with the technological advances to remain relevant, requiring significant effort in refactoring and implementation. Systems that easily adapt to new technologies will have a distinct advantage; in terms of software development strategies, agile software development will be more efficient than older waterfall-type models. Another area of improvement is that of algorithms and other aspects of methodology. With a strong connection to the R programming language, it is relatively easy to implement new algorithms in Breedbase, as they often require little modification from standalone scripts to work within Breedbase. At its core, Breedbase uses a relational database with integrated JSON data storage, which provides a healthy balance between highly structured, normalized data and flexibility. However, other systems, such as graph databases and highly parallelized solutions like Hadoop, or a combination thereof, are becoming popular and may be integrated into Breedbase in the future.

All of the Breedbase codes are open source and readily available on the code sharing site GitHub (https://github.com/solgenomics).

### Conclusions

Breedbase provides a fully open-source, scalable, and feature-rich breeding digital ecosystem that has been in use at the RTB crops breeding centers of the CGIAR for many years, starting with the NextGen Cassava database, Cassavabase (https://cassavabase.org/). The system has now been adopted by various breeding programs including vegetable and grain crops and maintains an open and collaborative approach to software development, allowing database customization for each research community while sustaining a common framework. Our hope is that Breedbase, and the digital ecosystem that it provides, can contribute, in a small way, to solving the world’s big problems with food scarcity and food quality, and thus contribute to improving subsistence farmers’ lives around the world.

## Web resources 

https://github.com/solgenomics/—Github repositories for Breedbase code, last accessed 4/18/2022

https://hub.docker.com/r/breedbase/breedbase#—Docker image for Breedbase server, last accessed 4/18/2022

https://breedbase.org/—Breedbase demo site, last accessed 4/18/2022

https://cassavabase.org/—Cassavabase, the flagship Breedbase site, last accessed 4/18/2022

https://musabase.org/—Breedbase site for banana breeding, last accessed 4/18/2022

https://yambase.org/—Breedbase site for yam breeding, last accessed 4/18/2022

https://sweetpotatobase.org/—Breedbase site for sweet potato breeding, last accessed 4/18/2022

https://www.youtube.com/channel/UC3jrvvzGKKEHzOriDBgnj0A—YouTube channel for Breedbase, last accessed 4/18/2022

## Data availability

All codes are available from Github (https://github.com/solgenomics) and docker hub (https://hub.docker.com/r/breedbase/breedbase#).
